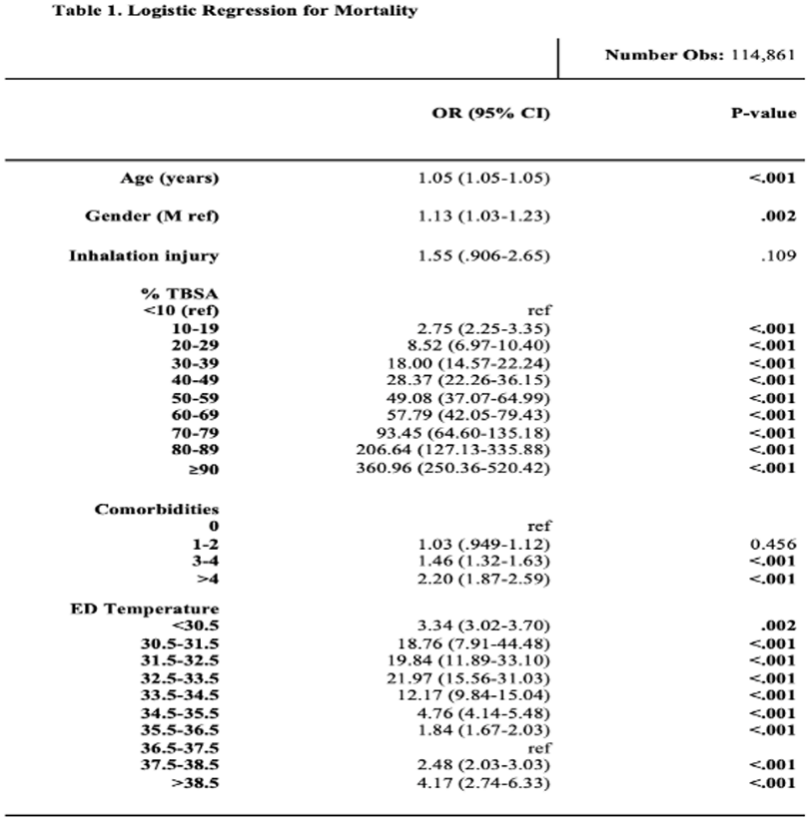# T5 Temperature Derangement on Admission Predicts Mortality in Burn Patients—a Nationwide Analysis and Opportunity for Improvement

**DOI:** 10.1093/jbcr/irad045.005

**Published:** 2023-05-15

**Authors:** Eloise Stanton

**Affiliations:** Keck School of Medicine of USC, Los Angeles, California

## Abstract

**Introduction:**

While single-institution studies have described the relationship between hypothermia, burn severity, and complications, there are no national estimates on how temperature on admission impacts hospital mortality. This study aims to evaluate the relationship between admission temperature and complications on a national scale to expose opportunities for improved outcomes.

**Methods:**

The US National Trauma Data Bank (NTDB) was analyzed between 2007-2018. Mortality was modeled using multivariable logistic regression including burn severity variables (% total burn surface area (TBSA), inhalation injury, emergency department (ED) temperature), demographics, and facility variables. Temperature was parsed into three categories: hypothermia ( < 36.0°C), euthermia (36.0-37.9°C), and hyperthermia (≥38.0°C).

**Results:**

116,796 burn encounters were included of which 77.9% were euthermic, 20.6% were hypothermic and 1.45% were hyperthermic on admission. For every 1.0C drop in body temperature from 36.0°C, mortality increased by 5%. Both hypothermia and hyperthermia were independently associated with increased odds of mortality when controlling for age, gender, inhalation injury, number of comorbidities, and %TBSA (p < .001). All temperatures below 36.0°C were significantly associated with increased odds of mortality. Patients with ED temperatures between 32.5-33.5°C had the highest odds of mortality across the cohort (22.0, 95% CI 15.6-31.0, p< .001).

**Conclusions:**

ED hypothermia and hyperthermia are independently associated with mortality even when controlling for known covariates associated with inpatient death. These findings underscore the importance of early warming interventions both at the prehospital stage and upon ED arrival. ED temperature could become a quality metric in benchmarking burn centers to improve mortality.

**Applicability of Research to Practice:**

This study provides national-scale data on the important relationship between ED temperature and mortality that can substantially impact both prehospital and hospital policies and practice and subsequently improve patient care.